# The social, economic, political, and genetic value of race and ethnicity in 2020

**DOI:** 10.1186/s40246-020-00284-2

**Published:** 2020-10-15

**Authors:** Tesfaye B. Mersha, Andrew F. Beck

**Affiliations:** 1 Division of Asthma Research, Cincinnati Children’s Hospital Medical Center, Department of Pediatrics, University of Cincinnati College of Medicine, 3333 Burnet Avenue, MLC 7037, Cincinnati, 45229-3016 OH USA; 2 Division of General and Community Pediatrics, Cincinnati Children’s Hospital Medical Center, Department of Pediatrics, University of Cincinnati College of Medicine, Cincinnati, OH USA; 3 Division of Hospital Medicine, Cincinnati Children’s Hospital Medical Center, Department of Pediatrics, University of Cincinnati College of Medicine, Cincinnati, OH USA

**Keywords:** Race, Ethnicity, Genetic ancestry, Health disparity, Human origin

## Abstract

Disparities across racial and ethnic groups are present for a range of health outcomes. In this opinion piece, we consider the origin of racial and ethnic groupings, a history that highlights the sociopolitical nature of these terms. Indeed, the terms race and ethnicity exist purely as social constructs and must not be used interchangeably with genetic ancestry. There is no scientific evidence that the groups we traditionally call “races/ethnicities” have distinct, unifying biological or genetic basis. Such a focus runs the risk of compounding equity gaps and perpetuating erroneous conclusions. That said, we suggest that the terms race and ethnicity continue to have purpose as lenses through which to quantify and then close racial and ethnic disparities. Understanding the root cause of such health disparities—namely, longstanding racism and ethnocentrism—could promote interventions and policies poised to equitably improve population health.

## Introduction

**Fig. 1:**
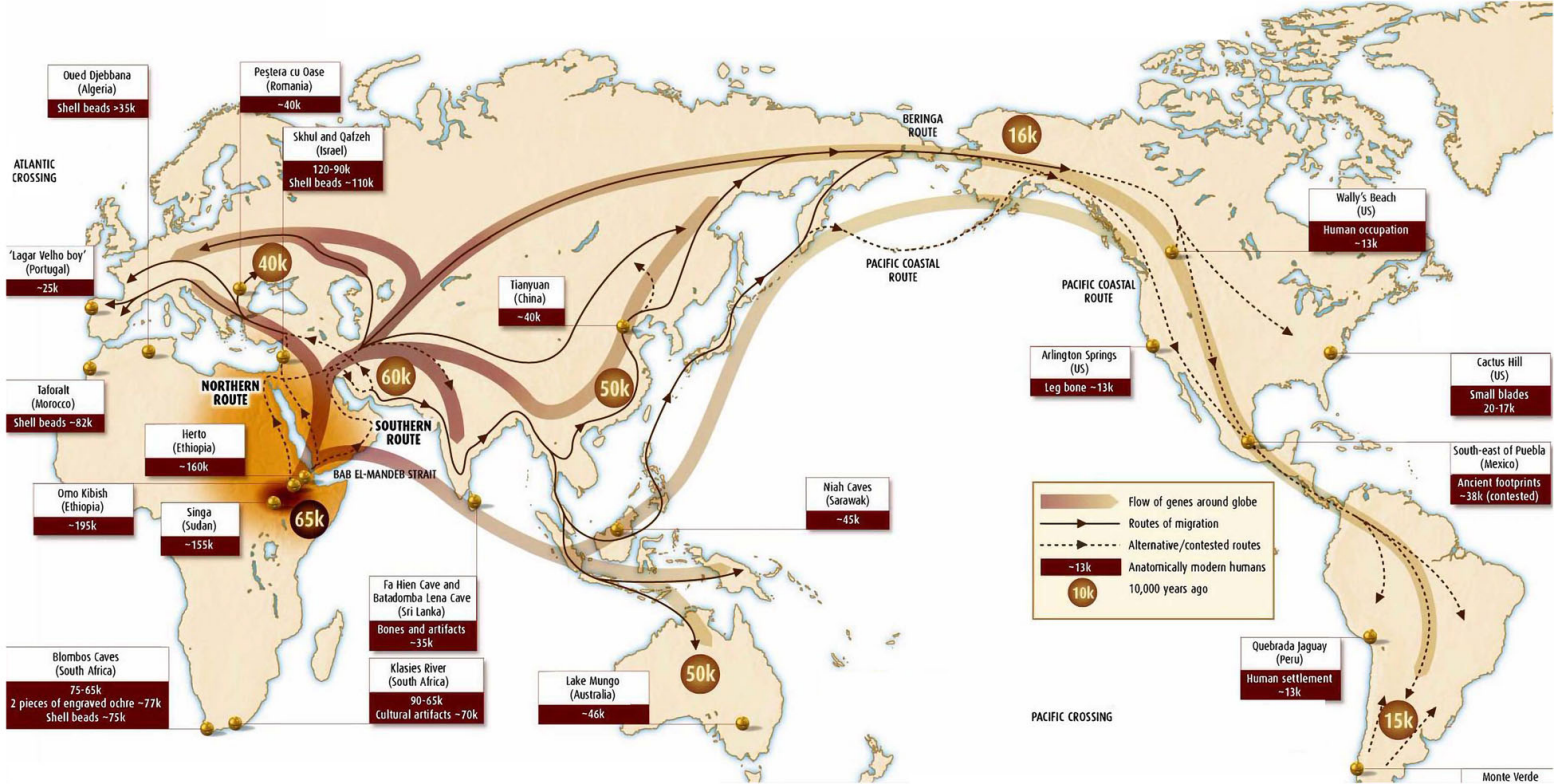
The origin of anatomically modern humans (*Homo sapiens*) and migration out of Africa. Fossil records, mitochondrial DNA analysis (mtDNA), Y chromosome, and nuclear genomic DNA shows that modern humans originated in Africa [1] and migrated out of African, then eventually to the rest of the world. Every mutation present in other parts of the world exist in Africa

DNA studies and archeological findings tell us that present-day humans descend from hominids in Ethiopia and surrounding territories. Early humans migrated “out of Africa” some two million years ago (Fig. 1). The final migration of modern humans out of Africa occurred ~ 60,000 years ago and led to the inhabitation of all continents except Antarctica [2]. Geographic separation and adaptation to local environmental conditions subsequently produced recognizable phenotypic variations in humans in different parts of the world. Despite phenotypic variation, underlying genotypes are remarkably similar [3]. Thus, in this opinion article, we argue that classification of race and ethnicity based on genetic information is not valid nor appropriate. It has been contextual factors and experiences stretching across history, and not genetics, that divided people into the racial and ethnic categories of today. In turn, it is racism and ethnocentrism that drive racial and ethnic-based health disparities.

## Historical perspectives on race and ethnicity

**Fig. 2:**
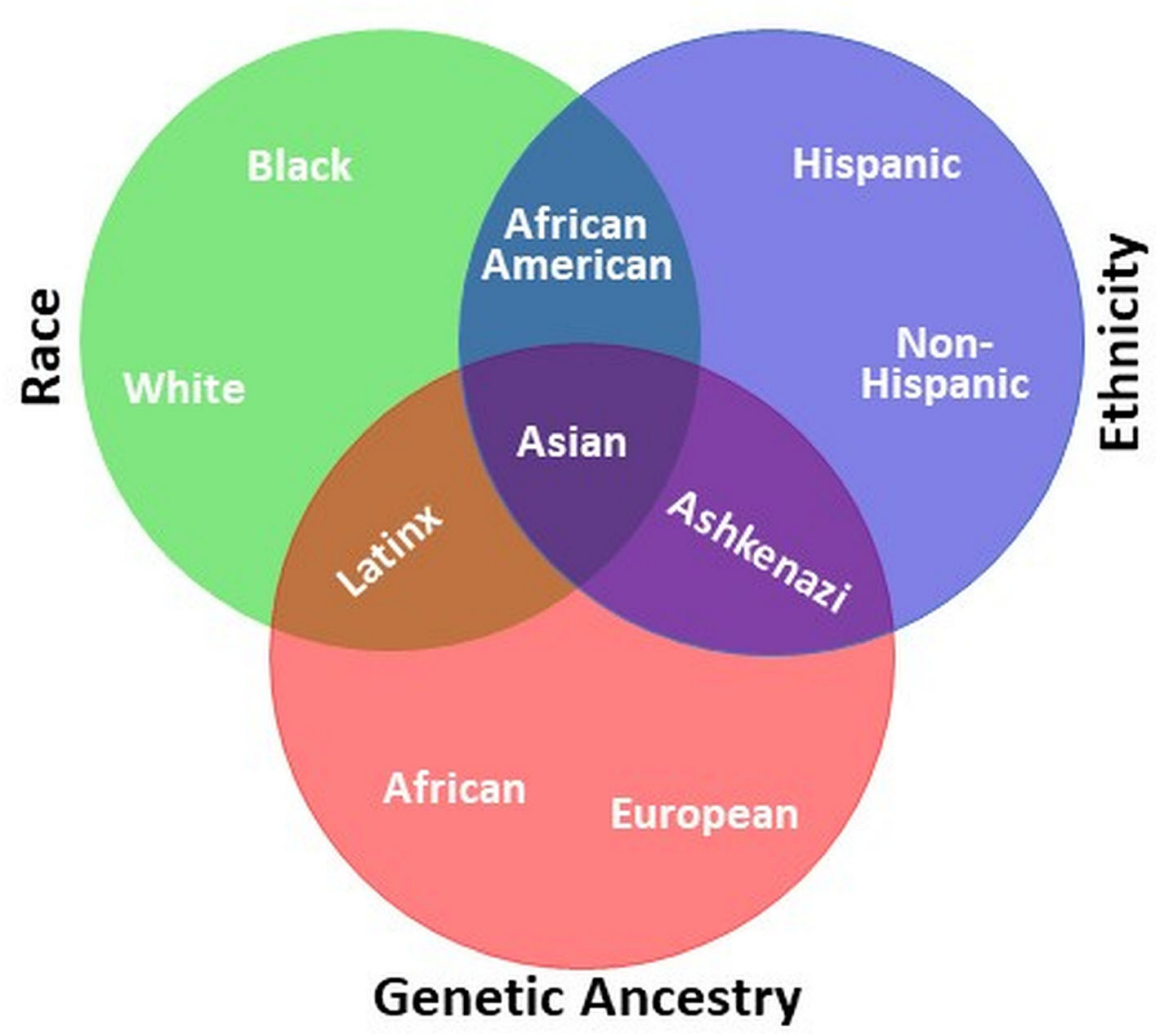
The use and relationship of racial, ethnic, and ancestral categories. Race is a social construct, ethnicity is a cultural construct often linked to community, religion, and language, and genetic ancestry is biological inheritance of DNA that can be traced through the genome. Human genetic diversity forms a continuum rather than discrete racial and ethnic clusters

Today’s racial and ethnic categories arose long before the field of genetics. The term “race” was first formally used in the English language around 1580, from the old French “rasse” and the Italian “razza,” to categorize modern humans. In the sixteenth and seventeenth centuries, scientists classified humans according to geographic locations using skin color, stature, and other distinguishing physical characteristics. Later, “nations” and “types” were introduced to describe humans living on different continents. Carl Linnaeus (1758), the Swedish biologist who developed the technique for classification of plants and animals, gave modern humans the scientific name *Homo sapiens*. Linnaeus further divided *Homo sapiens* into Europaeus (white skin), Asiaticus (yellow skin), Americanus (red skin), and Afer (black skin) on the basis of geography and skin color [4]. From this description, we see that even initial racial categories were built upon environmental and superficial physical characteristics. The concept of ethnicity emerged later, in the twentieth century, to replace and/or complement race. Ethnicity refers to shared culture, language, physical attributes, and religion. While race and ethnicity overlap, race calls on phenotypic attributes (skin tone and facial features almost exclusively) more so than ethnicity. Figure 2 displays relationships between concepts of race, ethnicity, and genetic ancestry which itself is defined according to biological inheritance of DNA and is traced through the genome-based line of decent. As used in the modern world, both race and ethnicity remain social constructs; neither term delineates genetic or biological categories [5].

## Humans more similar than different

Modern DNA sequencing technologies revolutionized our understanding of the human genome in ways that could not have been imagined before. We can now read all 3.2 billion nucleotides in a matter of hours. Scientists have investigated the DNA of thousands of people from around the world trying to find a genetic underpinning for racial or ethnic differences. They have found that human DNA is 99.9% similar, whether we are from Europe, Asia, the Americas, or Africa. In fact, there is more genetic diversity within a single racial/ethnic group than between two or more groups [5]. Two individuals in Africa can be more genetically dissimilar from each other than either one might be relative to an individual in Europe or Asia. To think that our racial or ethnic identities could be based upon a mere 0.1% of our genome and not our lived experience does not stand to reason, especially given that the small differences that do exist in our DNA are present to help us adapt to local environmental conditions.

Small existing genetic differences helped us adapt to local environmental conditions across the continents since the migration out of Africa [5]. This is true regardless of superficial physical characteristics, whether we are white or black, tall or short, brown-eyed or blue-eyed. Let us look at the following examples for how environment-specific conditions select certain alleles for local adaptation. Ancestors of modern humans lived and co-evolved with virus and other pathogens in an environment where infectious tropical diseases induced a strong immuno-genetic selection. Early in human evolution, parasite expulsion in the tropics, including malaria, was a dominant immunogenetic selective force in shaping human survival in Africa [6]. The Fulani people of West Africa have fewer malaria cases, lower prevalence of *Plasmodium falciparum* infection, and altered Th1/Th2 cytokine responses compared to their geographic neighbors. Sickle cell anemia (caused by hemoglobin-Beta gene mutations) and East African sleeping sickness (linked to the apolipoprotein L1 genetic variant; APOL1) gene represent two examples where environmental conditions selected some alleles for local adaptation as protection from indigenous diseases like malaria and Trypanosoma parasitism, respectively. However, these adaptations are not without physiologic consequences: sickle cell disease leads to painful crises and organ damage, and the APO1 variant is associated with increased susceptibility to chronic kidney disease (CKD). The APO1 variant, however, is missing among inhabitants of the Ethiopian highlands, where the Tsetse fly vector for the trypanosome parasite is absent, paralleling a very low incidence of CKD. Thus, small changes in our genetic makeup do occur, but they do so to adapt to environmental conditions. These genetic changes are not, however, race- or ethnicity-specific. Instead, changes can happen to any human population in the world in response to specific environmental conditions no matter their racial or ethnic categorization.

Trying to find a biological basis for differences across races or ethnicities is like looking for a needle in a haystack, akin to seeking a biological basis for why certain individuals and populations speak different languages. Our ability to speak a specific language, or more than one language, is not built upon our genes. Instead, language and culture are learned. They may be based on where we grow up or live; they can be altered and redefined [7]. Similarly, race and ethnicity exist in our minds and our social interactions, but not in our DNA. Assignment of genetic identity based on racial and ethnic affiliation is like slicing a soup: you can cut wherever you want, but the soup stays mixed. Racial and ethnic identities, and categories, change over time and place, and our ideas about race and ethnicity are shaped considerably by what surrounds us and not by what is inside of us.

## Genes do not cause racial/ethnic-health disparities

In the United States (US), there are marked racial and ethnic disparities with respect to a range of socioeconomic and environmental factors like poverty, educational attainment, access to healthcare, and proximity to pollutants [8]. Looking for a “purely genetical” explanation for differences in exposure to such factors and ensuing social or health outcomes does not do justice to the undeniable impact of racism and ethnocentrism. Instead, a focus on genes compounds, reinforces, and harmfully justifies racist and ethnocentric tropes.

To be clear, there is no evidence that genes drive racial and ethnic health disparities. Rather, racial disparities in health are better explained structural racism than by genetic differences. In the US, for every preventable disease, African Americans (AA) have the highest mortality rate and shortest expected years of life compared to non-Hispanic Whites [9–11]. This is true not because of DNA but because of differences in their lived experience, differences in the context in which individuals are “born, grow, work, live, and age” [12]. For instance, McKinnon et al. showed that Black women living in Canada have lower preterm birth (PTB) rates than Black/AA women living in the USA (8.9% vs 12.7%, respectively) [13]. Better birth outcomes among foreign-born Black women in the US compared to US-born Black/AA women [14], and COVID-19 case fatality rates that are lower among Africans in Africa than AA [15] further highlights that genetics cannot be used to explain health disparities; rather, it highlights again the importance of context. Indeed, this relationship between neighborhood context and health has been widely established [16]. As an example, in a study by Tessum and colleagues published in PNAS, AA individuals were exposed to 56% more pollution than they collectively contribute, and Hispanic individuals to 60% more pollution. Meanwhile, white individuals were exposed to 17% less pollution than what they contribute [17].

It is this context, and inequities therein, that drive between-group differences—disparities—in a range of health outcomes. We know that historically disadvantaged populations—including AA and Hispanic patients—have a higher burden of the comorbidities traditionally used by hospitals to stratify patients by risk. This is largely because of structural and socioeconomic factors. Studies and statistics suggest that, compared to their white counterparts, AA patients are 40% more likely to have high blood pressure, twice as likely to have heart failure, four times as likely to die from asthma-related complications, three times more likely to have chronic kidney disease, twice as likely to be diagnosed with colon and prostate cancer, and represent 44% of the HIV positive population. Across the US, we are seeing alarming statistics about the disproportionate toll of COVID-19 on AA and Latino people [11]. There has been a 21.6% increase in daily COVID-19 deaths in the counties with a higher population of AA residents vs. a 5% increase in counties with a higher population of White residents. To do the greatest good ethically and fairly, these persistent inequities must raise alarms.

These alarms, and the continued reality of racial and ethnic disparities, are real and require attention; they follow closely behind differences in the sociopolitical context that different racial and ethnic groups confront. There is plenty of evidence linking marginalization, segregation, and discrimination to a laundry list of adverse social, economic, environmental, and, yes, health outcomes. Race and ethnicity have been used to justify horrific discrimination across generations. This justification has been both explicit and implicit; it is at the root of the horrors we are now seeing in our streets. Indeed, George Floyd, Breonna Taylor, Ahmaud Arbery, Daniel Prude, Jacob Blake, and many more have borne the brunt of a classification schema built to perpetuate hierarchical tiers of status and power. These different tiers drive differences in the cleanliness of the air we breathe, the homes in which we live, and the resources we enjoy. Differences in health outcomes follow.

While there has been substantial effort in reconceptualizing the notion of racial disparities beyond genes/biology, the utility of the terms race and ethnicity remains problematic among scholars, the lay public, and other stakeholders. Genetic data can provide data on the nature of human biological diversity. How we incorporate this information into structural racism and race information will be critical in ensuring that modern understandings of human variation are viewed with anti-racist, anti-ethnocentric lenses. Yet, race and ethnicity must always be de-biologize and never be used interchangeably with genetics. Instead, they should be recognized as the sociopolitical [18] constructs that they are. In this way, we can use such categorizations, driven by self-report or self-identification, to monitor and, ultimately, narrow, longstanding equity gaps. By framing concepts of race and ethnicity in the context of racism and ethnocentrism, we can enumerate and then respond to the ramifications of generations of systemic, structural inequities from unjust policies and discrimination. We cannot eliminate disparities with medical treatments alone; instead, these social constructs must be confronted with social responses, with the pursuit of health (and social) justice.

## Conclusions

Homo sapiens evolved in Africa, and all humans indisputably share a common, unifying genetic origin. Genetic variation across Europe and Asia, and the Americas and Australia, is essentially a subset of the genetic variation in Africa. Regardless of differences in skin color, cultural background, religion, or language, we all are more similar than different! Genes do not cause disparities, rather systemic, structural inequities have long been the culprit. Just as one learns a new language through study and immersion, so too can we learn more about one another through social means, through interaction and advocacy for equity. Disparities did not emerge overnight nor are they amenable to simple, overnight solutions. Instead, we must recognize the context that underlies them, driven by sociopolitical and not genetic factors. Racial and ethnic disparities represent a multi-level, large scale problem in need of multi-level, large scale solutions [19].

## Data Availability

Not applicable, no datasets generated or analyzed.

## Abbreviations

AA: African American
DNA: Deoxyribonucleic acid
CKD: Chronic kidney disease
APO1: Apolipoprotein A1
Th1 cytokine: Th1-type cytokine
Th2 cytokine: Th2-type cytokine
