# Correction to: The social, economic, political, and genetic value of race and ethnicity in 2020

**DOI:** 10.1186/s40246-020-00292-2

**Published:** 2020-11-24

**Authors:** Tesfaye B. Mersha, Andrew F. Beck

**Affiliations:** 1 Division of Asthma Research, Cincinnati Children’s Hospital Medical Center, Department of Pediatrics, University of Cincinnati College of Medicine, 3333 Burnet Avenue, MLC 7037, Cincinnati, OH 45229-3016 USA; 2 Division of General and Community Pediatrics, Cincinnati Children’s Hospital Medical Center, Department of Pediatrics, University of Cincinnati College of Medicine, Cincinnati, OH USA; 3 Division of Hospital Medicine, Cincinnati Children’s Hospital Medical Center, Department of Pediatrics, University of Cincinnati College of Medicine, Cincinnati, OH USA

**Correction to: Hum Genomics 14, 37 (2020)**

**https://doi.org/10.1186/s40246-020-00284-2**

Following publication of the article [1], the authors identified errors in Fig. 2 and its caption. The corrected figure and caption can be found below. The corrections do not change the results or the conclusions of this article.

**Fig. 2:**
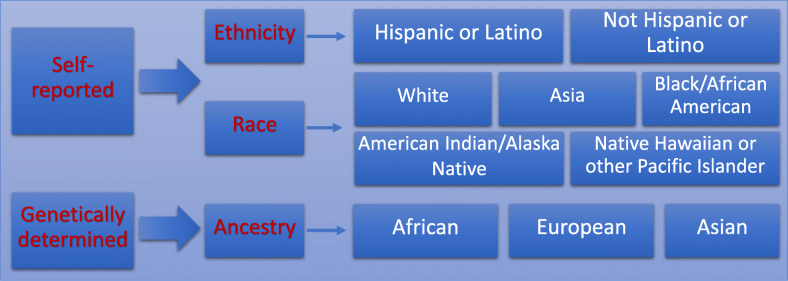
Major racial, ethnic and ancestry classification in the US (Modified from NIH Racial and Ethnic Categories and Definitions for NIH Diversity Programs and for Other Reporting Purposes. Notice Number: NOT-OD-15-089 (https://grants.nih.gov/grants/guide/notice-files/not-od-15-089.html))

The original article has been updated.
